# Hydrogen Bonding-Induced Assembled Structures and Photoresponsive Behavior of Azobenzene Molecule/Polyethylene Glycol Complexes

**DOI:** 10.3390/polym11081360

**Published:** 2019-08-16

**Authors:** Hsin-Tzu Tai, Yen-Chun Lin, Jing-Yao Ma, Chieh-Tsung Lo

**Affiliations:** Department of Chemical Engineering, National Cheng Kung University, No. 1, University Road, Tainan 701, Taiwan

**Keywords:** azobenzene, polyethylene glycol, hydrogen bonding, photoisomerization, crystallization

## Abstract

We investigated the self-assembled structures and photoresponsive and crystallization behaviors of supramolecules composed of 4-methoxy-4′-hydroxyazobenzene (Azo) molecules and polyethylene glycol (PEG) that were formed through hydrogen-bonding interactions. The Azo/PEG complexes exhibited the characteristics of photoresponse and crystallization, which originated from Azo and PEG, respectively. When Azo/PEG complexes were dissolved in solvents, hydrogen-bonding interaction hindered the rotation and inversion of mesogens, causing a reduction in the photoisomerization rate compared with the photoisomerization rate of the neat Azo. The confinement of Azo/PEG complexes in thin films further resulted in a substantial decrease in the photoisomerization rate but an increase in the amounts of H-aggregated and J-aggregated mesogens. Regarding PEG crystallization, ultraviolet irradiation of Azo/PEG complexes increased the quantity of high-polarity cis isomers, which improved the compatibility between mesogens and PEG, subsequently increasing the crystallization temperature of PEG. Moreover, the complexation of Azo and PEG induced microphase separation, forming a lamellar morphology. Within the Azo-rich microphases, mesogens aggregated to form tilted monosmectic layers. By contrast, PEG crystallization within the PEG-rich microphases was hard confined, indicating that the domain size of the lamellar morphology was unchanged during PEG crystallization.

## 1. Introduction

The molecular design and synthesis of multifunctional polymeric materials have received much attention because of their favorable properties in a variety of fields [1,2,3,4,5,6]. Among them, photoresponsive polymers comprising azobenzene chromophores are of great scientific interest because of their potential application in optical data storage, holographic recording, and surface-relief gratings [7,8,9,10,11]. Photoinduced properties of azobenzene-containing polymers originate from the photoisomerization of azobenzene mesogens. When azobenzene-containing polymers are irradiated with ultraviolet (UV) light, the trans-to-cis isomerization of mesogens is triggered. Because cis isomers have a higher energy level than trans isomers do, cis isomers spontaneously convert to trans isomers in dark and heated conditions. Additionally, cis-to-trans isomerization can be induced with visible light irradiation. Photoisomerization simultaneously changes the molecular shape of mesogens, which in turn alters the dipole moments of azobenzene-containing materials [12]. When rigid azobenzene mesogens are connected to the backbone of flexible polymer chains, the resulting azobenzene-containing polymers exhibit liquid-crystalline characteristics, with azobenzene units forming mesophases. The alignment of azobenzene mesogens in mesophases may be achieved by applying linearly polarized light [13,14,15,16]. Furthermore, the absorbance of azobenzene-containing polymers strongly depends on the aggregation state of azobenzene chromophores. The parallel-packing arrangement of the mesogens (H-aggregation) causes a blue shift of the absorption maximum compared with the absorption maximum of nonassociated mesogens, whereas the head-to-head organized mesogens (J-aggregation) result in a red shift of the absorption maximum [17].

Synthetic methods for preparing azobenzene-containing polymers with well-defined photochemical and photochemical properties have been reported extensively [16,18,19,20,21,22,23,24,25,26]. Although these approaches provide precise control of theses polymers′ molecular structures, the multistep synthetic routes are costly in terms of both time and money. Currently, supramolecular concepts are used to prepare polymers that carry azobenzene moieties [27,28,29,30,31,32,33]. These concepts rely on the complexation of polymers and chromophores through noncovalent intermolecular interactions such as hydrogen bonding and ionic bonding, allowing for easy variation of the content and type of azobenzene derivatives and easy design of materials functionality. Depending on the balance between the association interactions and chromophore–chromophore interactions, these molecules self-assemble into large functional units and structural hierarchies, resulting in functional materials with unique photochemical and photophysical properties. For instance, Priimagi et al. created a complex of various homopolymers with azobenzene molecules through hydrogen-bonding interaction [28,29]. Hydrogen-bonding interaction prevented chromophores from aggregating, thereby enhancing the photoinduced anisotropy of the complexes. Additionally, because of hydrogen bonds, the reduced mobility of azobenzene mesogens improved the temporal and thermal stability of photoinduced birefringence. Furthermore, adding a large quantity of chromophores to homopolymers induced a smectic-like self-organization of azobenzene mesogens, whereas complexes with fewer chromophores lost the association of chromophores, resulting in a disordered structure [33]. The isomerization rate of complexes with self-assembled mesogenic units was much higher than that of complexes with a disordered structure. Some researchers prepared complexes composed of polystyrene-block-poly(4-vinylpyridine) (PS-b-P4VP) diblock copolymers and carboxy-terminated azobenzene derivatives [31]. The complexes exhibited hierarchical structures, including ordered phases through microphase separation of PS-b-P4VP and mesophases composed of chromophores embedded in the P4VP microdomains. The morphology of the complexes strongly affected the photoinduced birefringence. Complexes with a spherical structure exhibited lower-order parameters than those with a lamellar structure did.

In this study, we systematically investigated the structure and photoresponsive behavior of supramolecular polymers containing azobenzene. Polyethylene glycol (PEG) was selected as a constituent because introducing PEG as one constituent endowed supramolecular polymers with hydrophilicity, ion conductivity, and crystallization [34,35,36,37], thus offering greater possibilities in terms of their functional and structural diversities. This study clearly demonstrated the effect of the complex composition in altering the aggregation state of mesogens and the phase morphologies of the complexes. Although a few reports have addressed the behavior of azobenzene/polymer complexes, literature on the microstructure of complexes composed of semicrystalline polymers is scarce. We believe that this paper provides valuable data that complement the literature and may provide deep insights into the structure and photoresponsive behavior of supramolecular materials containing azobenzene.

## 2. Materials and Methods

**Figure 1:**
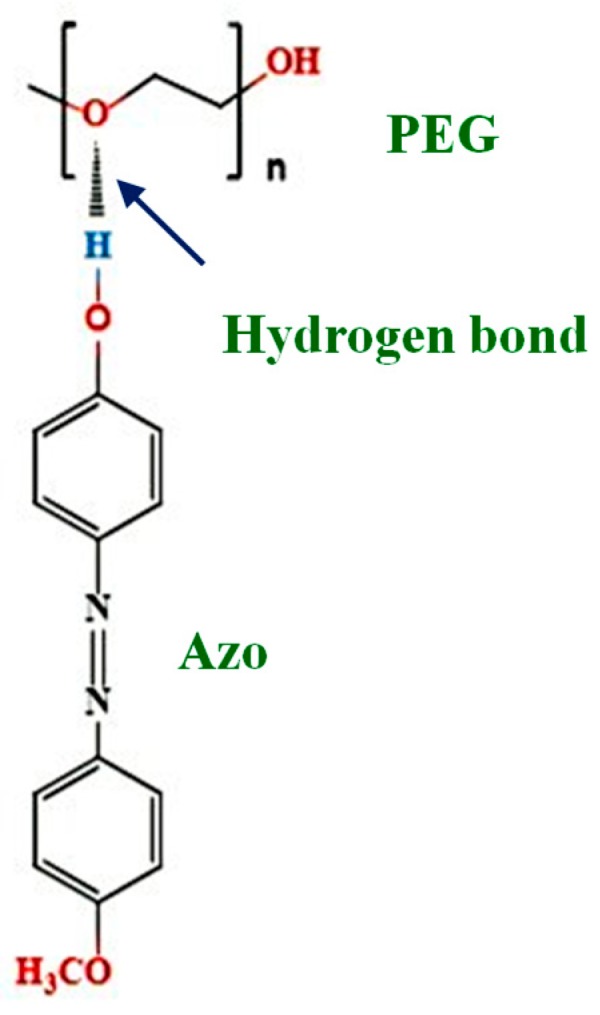
Chemical structures of polyethylene glycol (PEG) and 4-methoxy-4′-hydroxyazobenzene (Azo).

The compound *p*-anisidine (99%) and phenol (99%) were obtained from Alfa Aesar (Tewksbury, MA, USA) and used as received. Hydrochloric acid (HCl, 36.5–38%), toluene (99.6%), and dichloromethane (CH_2_Cl_2_, 99.8%) were purchased from J. T. Baker (Radnor, PA, USA) and used without further purification. Sodium nitrite (NaNO_2_, 99%), benzene (99.9%), and poly(ethylene glycol) methyl ether (PEG) with a number-averaged molecular weight (*M*_n_) of 5000 g/mol and a polydispersity index of 1.06 were provided by Sigma-Aldrich Co. (St. Louis, MO, USA) The chemical structure of PEG can be seen in Figure 1. Sodium hydroxide (NaOH, 97%) was obtained from Showa Corporation (Saitama, Japan). Hexane was purchased from Tedia (Fairfield, OH, USA). Tetrahydrofuran (THF, 99.3%) was provided by Echo Chemical Corporation (Miaoli, Taiwan).

The azobenzene monomer 4-methoxy-4′-hydroxyazobenzene (Azo) was synthesized according to a documented method [38]. Briefly, 73 mmol *p*-anisidine was added to 300 mL of 2 M HCl, and the solution was stored in an ice bath. Subsequently, 72.5 mmol Na_2_NO_3_ dissolved in 150 mL of deionized water was added dropwise to the solution. The 73 mmol phenol was dissolved in 200 mL of 2 M NaOH, and the solution was maintained at 0 °C. Subsequently, the *p*-anisidine and phenol solutions were mixed, and diazotization of *p*-anisidine with phenol was conducted for 2 h at room temperature. The precipitate was recrystallized using a 7:2 (*v*/*v*) mixed solution of hexane and benzene to obtain Azo (Figure 1). Proton nuclear magnetic resonance was as follows: δ (CDCl_3_) 3.9 (s, 3H, ArOCH_3_), 5.6 (s, 1H, –OH), 6.9–7.9 (m, 8H, aromatic). Molecular formula was as follows: C_13_H_12_N_2_O_2_.

The Azo/PEG complexes were prepared by mixing known quantities of both materials in THF. These mixtures were stored in ambient conditions for several days. The polymer solutions were then cast on Teflon cloth to prepare bulk specimens. The residual solvent was removed by maintaining the complexes at 80 °C in a vacuum oven for 1 day. For thin films of complexes, a total mass of 40 mg Azo/PEG was dissolved in 2 mL of THF. The complex solution was spin coated on the quartz cell at a spin rate of 250 rpm.

Fourier transform infrared (FTIR) spectra were obtained using a Thermo Scientific Nicolet 6700 spectrometer (Thermo Fisher Scientific Inc., Waltham, MA, USA) at a resolution of 2 cm^−1^. The UV spectroscopy of complex solutions was conducted using a Perkin Elmer Lamba 25 spectrophotometer (Perkin Elmer, Waltham, MA, USA). Complex solutions were prepared by dissolving Azo and PEG in various solvents at a concentration of 4 × 10^−5^ mol Azo/L. UV illumination was applied to the complex solutions at a wavelength of 365 nm and an intensity of 4.5 mW/cm^2^, and visible light irradiation was applied at a wavelength of 430 nm and an intensity of 4.3 mW/cm^2^. Optical microscopy was performed with a Nikon H550L Eclipse 50i LV-UEPI microscope (Nikon, Tokyo, Japan) in conjunction with a hot stage. The thermal properties of Azo/PEG complexes were characterized using a differential scanning calorimeter (DSC 7, Perkin Elmer, Waltham, MA, USA) under constant nitrogen flow. Temperature and heat flow were calibrated with indium and tin standards. Samples were sealed in the aluminum pan. Prior to measurements, samples were heated to 70 °C for 10 min. The samples were then cooled to 0 °C at a cooling rate of 5 °C/min to induce crystallization. Subsequently, the samples were reheated to above their melting temperatures at a heating rate of 5 °C/min.

Simultaneous small-angle X-ray scattering and wide-angle X-ray scattering (SAXS/WAXS) measurements were performed at Sector 23A1 at the National Synchrotron Radiation Research Center in Taiwan. Samples for SAXS/WAXS measurements were prepared by encapsulating complexes between two Kapton sheets. The sample-to-detector distance was 2 m, and the X-ray energy and wavelength were 10 keV and 1.24 Å, respectively. Initially, samples were heated to 70 °C, exceeding the melting temperature of PEG, and maintained at that temperature for 10 min to erase residual crystals. Subsequently, samples were cooled to 0 °C at a cooling rate of 5 °C/min to allow for PEG crystallization. During nonisothermal crystallization, both SAXS and WAXS patterns were collected simultaneously at 1-min intervals. For the SAXS measurements, the scattering data were appropriately corrected for incident flux, absorption, detector sensitivity variation, and dark current, and the scattering intensity was azimuthally averaged to obtain the one-dimensional intensity data [*I*(*q*)] as a function of the scattering vector (*q*), where *q* = 4·*π*·sin*θ*/*λ*, *θ* is the half-scattering angle, and *λ* is the incident radiation wavelength. Furthermore, the SAXS patterns of complexes at the low *q* regions (0.4–0.85 Å^−1^) were obtained using a Bruker diffractometer (NanoSTAR U System, Bruker AXS Gmbh, Karlsruhe, Germany), operated with a filtered CuKα radiation source.

## 3. Results and Discussion

**Figure 2:**
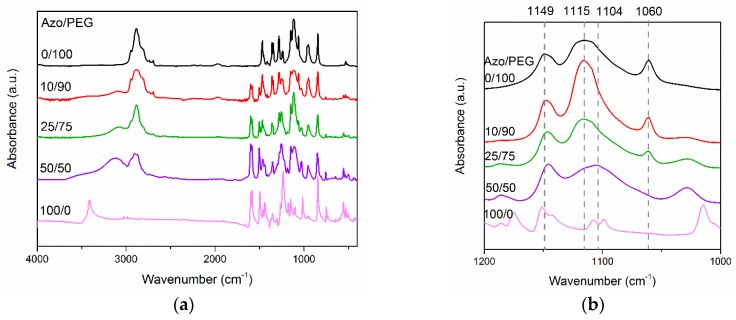
Fourier transform infrared (FTIR) of Azo/PEG complexes at wavenumbers of (**a**) 4000–400 cm^−1^ and (**b**) 1200–1000 cm^−1^. The complex compositions are designated in wt %.

Figure 2 depicts the FTIR spectra of Azo/PEG complexes with various Azo contents, which are presented in weight percentage. For the neat Azo, the absorbance at a wavenumber of 3412 cm^−1^ corresponded to the O–H stretch. The sharp peak was an indication that almost no hydrogen bonds existed between Azo molecules because of steric hindrance. When Azo was added to PEG, the absorbance of the O–H stretch shifted to a wavenumber of approximately 3105 cm^−1^ with a broad feature, indicating that most of the hydroxyl groups in Azo formed hydrogen bonds with the ether groups in PEG, as seen in Figure 1. Additionally, when the amount of Azo in Azo/PEG complexes increased, the absorbance at a wavenumber of 1115 cm^−1^ shifted to a wavenumber of 1104 cm^−1^. Such a shift usually occurs when ether groups exhibit intermolecular interactions with other molecules [39,40]. The intermolecular interaction in this study was attributed to hydrogen-bonding interaction. Quantitative correlations between the complex composition and hydrogen-bonding strength are difficult using the spectra because the wavenumber shift/enthalpy correlations are system dependent [41,42]. However, the wavenumber shift increased approximately with increasing Azo content, which was associated with an increase in the population of hydrogen bonding between PEG and Azo. Furthermore, the absorbance at a wavelength of 1115 cm^−1^ was associated with the noncrystalline C–O–C stretch in PEG. By contrast, the absorbance at wavelengths of 1149 and 1060 cm^−1^ corresponded to the crystalline C–O–C stretch in PEG [43]. The absorbance at a wavenumber of 1060 cm^−1^ decreased considerably with an increase in the Azo content, suggesting that the addition of Azo hindered PEG crystallization.

**Figure 3:**
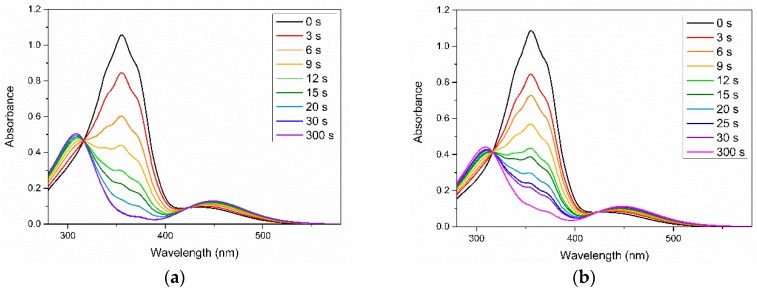
Variations in the UV–Vis spectra of (**a**) neat Azo and (**b**) 50:50 Azo/PEG complex dissolved in benzene during UV irradiation. The complex compositions are designated in wt %.

The most intriguing characteristics of azobenzene molecules is their photoinduced response after UV irradiation. Figure 3 presents the variations of the UV–Vis spectra of the Azo and 50:50 Azo/PEG complex in benzene upon UV irradiation. Initially, maximum absorbance was observed at a wavelength of approximately 360 nm for both samples regardless of the addition of PEG. This absorbance was associated with the *π*–π* absorption of trans isomers. When the Azo and Azo/PEG complex were exposed to UV light, the absorbance at a wavelength of 360 nm decreased considerably. Simultaneously, we observed an increase in the absorbance at a wavelength of approximately 450 nm, which corresponded to the *n*–π* absorption of cis isomers. Although the entire curve of the UV spectra changed with isomerization, two absorbance values at wavelengths of approximately 320 and 425 nm remained constant. The presence of these two isosbestic points indicated that the two distinct absorbing species equilibrated with each other and that no side reaction occurred during photoisomerization [44]. Furthermore, the *π*–π* absorption of trans isomers decreased consistently as UV exposure time increased until a photostationary state was achieved. Varying solvents did not influence the photoresponsive characteristic of the Azo and Azo/PEG complexes but changed the time required to reach the photostationary state. The first-order kinetics were assumed in research to describe the kinetics of the trans-to-cis photoisomerization of azobenzene mesogens, as given by [45]:

ln(*A*) = −*k·t* (1)

where *A* = (*A*_∞_ − *A*_t_)/(*A*_∞_ − *A*_o_); *A*_∞_, *A*_t_, and *A*_o_ are the absorbance values at the photostationary state, after UV irradiation for time *t*, and before UV irradiation, respectively, and *k* is the rate constant. Table 1 summarizes the rate constants obtained from the slope of the linear fit in various solvents. When the Azo/PEG complex was dissolved in benzene, toluene, and CH_2_Cl_2_, the photoisomerization rate of the Azo/PEG complex decreased compared with that of the neat Azo in these solvents. Such behavior was attributed to the hydrogen-bonding interaction between Azo and PEG, which hindered the rotation and inversion of azobenzene molecules, thereby reducing the photoisomerization rate of the complex. By contrast, when the complex was dissolved in THF, the photoisomerization rate was unchanged. This was presumably caused by the formation of the hydrogen bonds between the ether groups in THF and the hydroxyl groups in Azo that limited the formation of the Azo/PEG complex. Consequently, the photoisomerization rate of the Azo/PEG complex in THF was not retarded.

Another study indicated that the wavelength of the maximum absorbance depends on the aggregation state of azobenzene mesogens [17]. Nonassociated azobenzene mesogens resulted in maximum absorbance at 360 nm. By contrast, H-aggregated and J-aggregated azobenzene mesogens caused a blue and red shift of the maximum absorbance to the wavelengths of 334 and 384 nm, respectively. In this study, the neat Azo and Azo/PEG complex in various solvents exhibited the maximum absorbance at approximately 360 nm, indicating that most azobenzene mesogens were not aggregated. In these systems, all the solvents are good solvents for both Azo and PEG. Thus, azobenzene molecules in the neat Azo and Azo/PEG complex were not confined in an environment such as micelles or vesicles. Instead, they formed a relaxed conformation. Consequently, the aggregation of azobenzene mesogens was severely hindered, and most mesogens were not associated.

**Figure 4:**
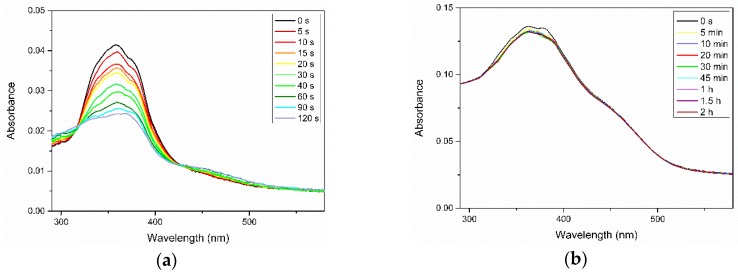
Variations in the UV–Vis spectra of (**a**) neat Azo and (**b**) 50:50 Azo/PEG complex in thin films during UV irradiation. The complex compositions are designated in wt %.

We also examined the photoisomerization of Azo and its complexes with PEG in thin films. Similar to the photoresponsive behavior in solvents, the transition from trans to cis isomers occurred in the neat Azo and Azo/PEG thin films exposed to UV light but with a noticeable broadening and blue shifts of maximum absorbance, as seen in Figure 4. Additionally, the change in the maximum absorbance from its initial state to the stationary state in thin films was much slower compared with that in solvents, indicating that the confined environment suppressed the photoisomerization of azobenzene mesogens. We further extended the UV exposure time but did not observe any marked changes in the absorbance. The photoisomerization rate obtained for the neat Azo thin film was 0.028 s^−1^, which was nearly one order of magnitude lower than that in solvents. Incorporating Azo into PEG further reduced the photoisomerization rate to 0.0043 and 0.0033 s^−1^ for the complexes containing 50% and 25% Azo, respectively. In addition to the confinement effect, the reduced kinetics of photoisomerization were caused by the slow dynamics of PEG in the solid state.

**Figure 5:**
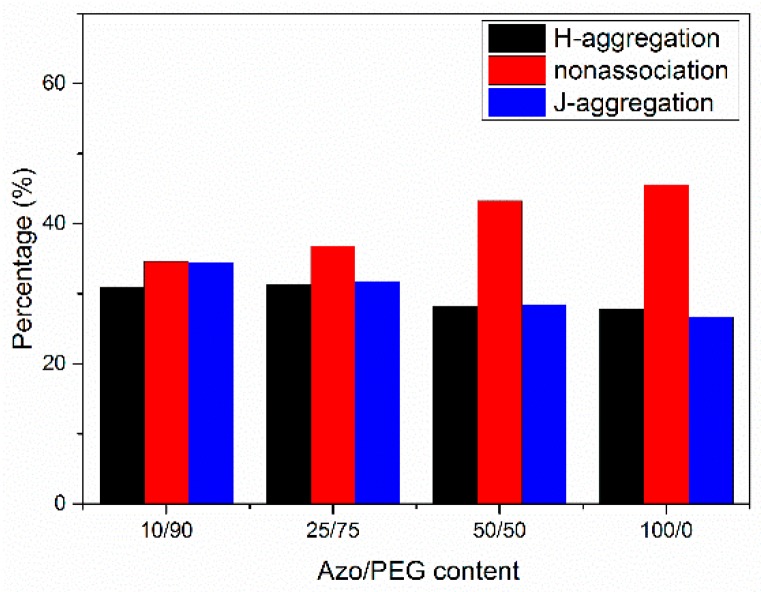
The populations of different organizations of azobenzenes in Azo/PEG complexes with regard to complex compositions.

We deconvoluted the UV spectra of Azo/PEG complexes in thin films according to the wavelengths of H-aggregated, nonassociated, and J-aggregated mesogens at 334, 360, and 384 nm, respectively. The area of each deconvoluted peak divided by the total area of the absorbance gives the proportion of the different aggregation states for azobenzenes, the results are presented in Figure 5. In the neat Azo, nonassociated mesogens dominated. When Azo formed complexes with PEG, the proportion of nonassociated mesogens decreased monotonically, whereas the proportions of J-aggregated and H-aggregated mesogens increased with increasing PEG content. Such behavior indicated that the confined domains generated by the self-assembly of Azo and PEG facilitated the organization of azobenzene molecules. Studies have suggested that when azobenzene molecules are confined, such as in azobenzene-containing homopolymers, in microphase-separated domains of block copolymers, or in the core of micelles, mesogens tend to assemble into H-aggregation [23,25,26,46]. Although the self-assembly of azobenzene molecules and PEG through hydrogen bonds was unable to form compact microdomains to heavily turn nonassociated mesogens into aggregated mesogens, as micelles and block copolymers do, the formation of Azo/PEG complexes could nonetheless facilitate the aggregation of mesogens. This finding suggested that intermolecular interaction enables manipulation of the azobenzene molecules′ aggregation state.

**Figure 6:**
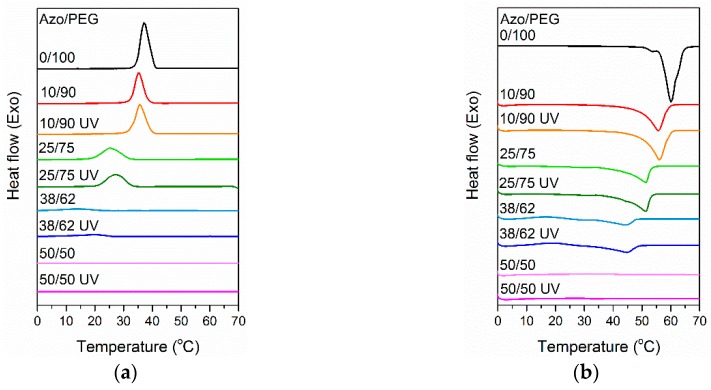
Differential scanning calorimeter (DSC) thermograms of Azo/PEG complexes during (**a**) nonisothermal crystallization and (**b**) reheating. The complex compositions are designated in wt %.

To understand the effect of photoisomerization on the crystallization behavior of Azo/PEG complexes, UV-exposed and non-UV-exposed Azo/PEG complexes were characterized using DSC. Figure 6a compares the heat flow of Azo/PEG complexes with and without UV exposure during cooling. Table 2 summarizes the crystallization temperature of the complexes extracted from the DSC curves. Generally, the blend system with the presence of hydrogen bonds exhibits a lower critical solution temperature behavior. High temperatures weaken hydrogen-bonding interaction, leading to the phase separation of the blend. However, hydrogen-bonded hydroxyl groups and ether groups normally dissociate at temperatures much higher than 100 °C [42,47]. Therefore, the experimental conditions used in this study would not cause the dissociation of PEG and Azo.

In Table 2, adding Azo to PEG considerably decreased the crystallization temperature of PEG. PEG crystallization was prevented when the Azo content was 50%. The decrease in the crystallization temperature upon the addition of Azo was attributed to dilution of the crystallizable chains of PEG when Azo was added to PEG. Additionally, the rigid chain structure of Azo acted as an obstacle for PEG diffusion. Consequently, the nucleation of PEG crystals was hindered, causing the crystallization temperature to decrease.

When the nonisothermal crystallization of Azo/PEG complexes with and without UV exposure was compared, UV exposure caused an increase in the crystallization temperature of PEG. The change in crystallization temperature was more pronounced when more Azo was incorporated into PEG. When Azo/PEG complexes were exposed to UV light, trans isomers converted to cis isomers through photoisomerization. Cis isomers exhibited higher polarity than trans isomers, thereby improving the compatibility between azobenzene mesogens and PEG. Such behavior facilitated the PEG chain mobility, thus increasing the crystal growth rate. Furthermore, the rod-shaped structure of trans isomers with a large radius of gyration disturbed PEG diffusion to the crystal growth front more than the bent structure of cis isomers with a small radius of gyration. Consequently, the crystallization temperature of Azo/PEG complexes with UV irradiation was higher than those without UV irradiation.

We reheated the Azo/PEG complexes to exceed the melting temperature of PEG, as shown in Figure 6b. The melting temperature of the Azo/PEG complexes decreased as the Azo content increased (Table 2). The depression of the melting temperature of PEG with the addition of Azo was attributed to the presence of mesogens, which prevented the growth of well-developed lamellar crystals. Furthermore, adding Azo to PEG caused a substantial decrease in the degree of crystallinity of PEG, which was caused by the hydrogen bonding between PEG and Azo that suppressed PEG crystallization. When the Azo/PEG complexes were exposed to UV light, the resultant melting temperature and degree of crystallinity were nearly unchanged compared with those of the complexes without UV irradiation. The melting behaviors suggested that the free energy of folded-chain crystals was nearly identical for cis isomers with bent structures and trans isomers with rod-shaped structures.

**Figure 7:**
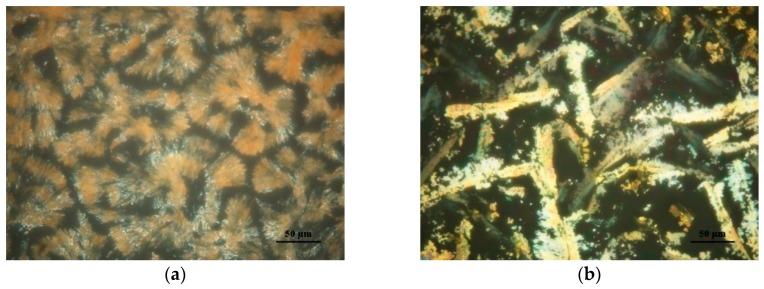
Polarized optical microscopy of (**a**) Azo at 60 °C, (**b**) 75:25 Azo/PEG at 60 °C. The complex compositions are designated in wt %.

Figure 7 illustrates the polarized optical microscopy of Azo and its complexes with PEG. At temperatures below the melting temperature of PEG, the presence of PEG crystals made the identification of liquid-crystalline (LC) textures formed by mesogens difficult. Therefore, we only conducted optical microscopy observation at temperatures higher than 60 °C, at which temperature PEG was amorphous in all the complexes. The neat Azo exhibited the typical focal conic-fan shaped texture at 60 °C (Figure 7a), suggesting a smectic LC phase. During heating, the color caused by the birefringence did not change and the texture remained the same. When the temperature reached 80 °C, the LC-to-isotropic phase transition occurred. In the complex containing 75% Azo, the sample also displayed the focal conic texture (Figure 7b). However, the nonbirefringent domains increased compared with those in the neat Azo. Similarly, the LC-to-isotropic phase transition occurred at 80 °C. When the Azo content decreased to 25%, the birefringence resulting from liquid crystals disappeared, indicating that the LC feature of the complex was overwhelmed by PEG.

**Figure 8:**
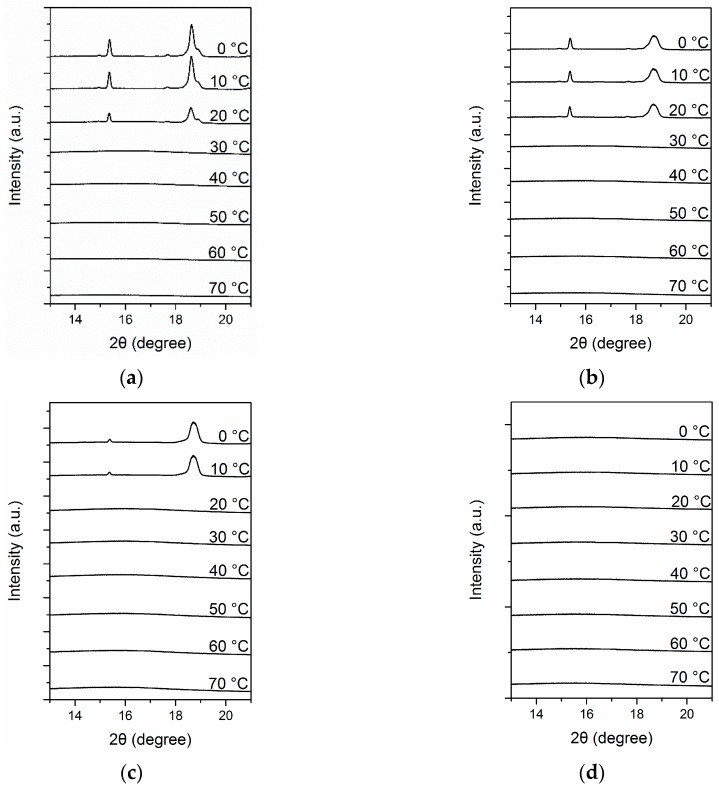
Time-resolved wide-angle X-ray scattering (WAXS) patterns of PEG and its complexes with Azo during nonisothermal crystallization at a cooling rate of 5 °C/min. (**a**) PEG, (**b**) 10:90 Azo/PEG, (**c**) 25:75 Azo/PEG, and (**d**) 50:50 Azo/PEG. The complex compositions are designated in wt %.

As discussed in preceding sections, the aggregation states of complexes containing azobenzene strongly depended on the structure of the complexes. The aggregation state of mesogens further determined the photoresponsive behavior of complexes. However, the specific crystal morphology and crystallization behavior of PEG could influence the final properties of the complexes, such as optical properties and adhesion. Therefore, we examined structural changes in Azo/PEG complexes using simultaneous SAXS and WAXS. Figure 8 presents the WAXS patterns of neat PEG and its complexes with Azo during cooling. For the neat PEG (Figure 8a), when the temperature exceeded 30 °C, only an amorphous halo was observed. When the temperature decreased to lower than 30 °C, two diffraction peaks at 2θ of 15.4° and 18.5° developed and were assigned as the (120) and (112/004) crystalline planes, respectively [48]. The addition of Azo did not alter the crystal structure of PEG. However, the onset crystallization temperature gradually decreased as the Azo content increased, indicating that the addition of Azo resulted in a decrease in the nucleation rate of PEG. This is consistent with the DSC results. Furthermore, adding Azo to PEG reduced the peak intensity and increased the peak width, indicating that the crystal structure of PEG tended to be more imperfect in the complexes. When the Azo/PEG content was 50:50 (Figure 8d), all the diffraction peaks disappeared, suggesting that no crystallization occurred.

**Figure 9:**
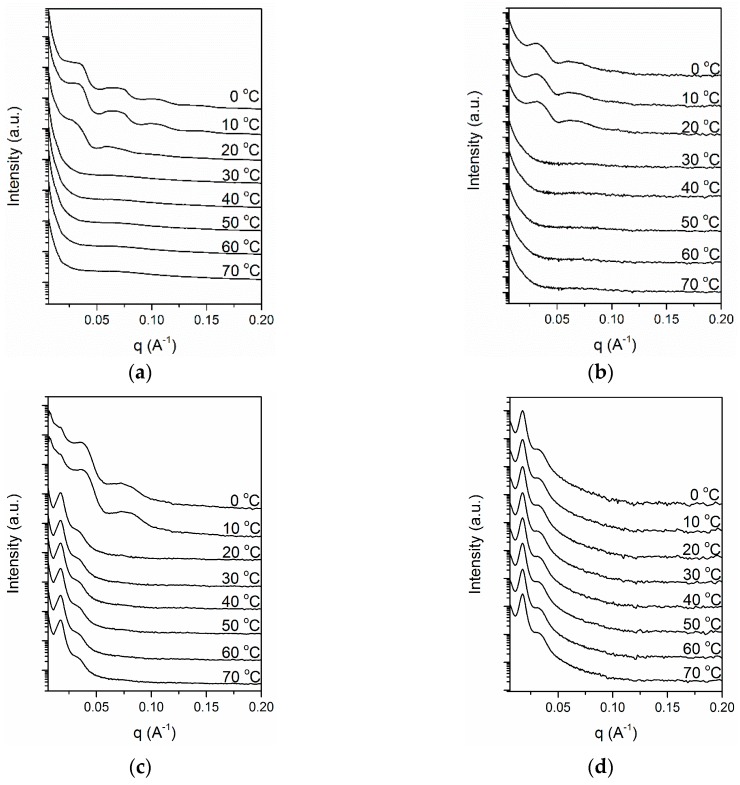
Time-resolved small-angle X-ray scattering (SAXS) patterns of PEG and its complexes with Azo during nonisothermal crystallization at a cooling rate of 5 °C/min. (**a**) PEG, (**b**) 10:90 Azo/PEG, (**c**) 25:75 Azo/PEG, and (**d**) 50:50 Azo/PEG. The complex compositions are designated in wt %.

Figure 9 depicts the series of SAXS patterns of the neat PEG and its complexes with Azo during cooling. The SAXS pattern of the neat PEG, as portrayed in Figure 9a, exhibited multiple peaks at *q* values of 0.035, 0.070 and 0.106 Å^−1^, which resulted from the crystalline-amorphous structure of PEG crystals. The broad feature of these peaks was caused by the nonuniform thickness of chain-folded lamellae. The long period of the crystal lamellar structure, calculated using Bragg’s law, was 18.0 nm. The peaks began to appear at 20 °C, which is consistent with the onset crystallization temperature obtained in WAXS, confirming that the peaks in the SAXS pattern originated from PEG crystallization. Similar crystallization behavior was observed when the Azo/PEG content was 10:90 (Figure 9b). When the Azo content was increased to 25% (Figure 9c), two sharp peaks were observed at *q* values of 0.017 and 0.034 Å^−1^ at 70 °C. At this temperature, PEG was amorphous, therefore, the two peaks were not caused by PEG crystallization. We attributed these peaks to the microphase separation of PEG and Azo. The peaks exhibited the *q* ratio of 1:2, indicating that PEG and Azo microphase separated to a lamellar morphology. During cooling, the morphology of the Azo/PEG complex was retained and the domain size remained constant. When the temperature reached 10 °C, PEG crystallization occurred, confirmed by the development of two broad peaks located at *q* values of 0.037 and 0.074 Å^−1^. Although the intensity of the diffraction peaks originating from the microphase separation of Azo and PEG was overwhelmed by the intensity of the diffraction peaks contributed by PEG crystallization, the presence of a small peak at the q value of 0.017 Å^−1^ suggested that Azo and PEG still exhibited microphase separation. These results indicated that two types of structures with different length scales, including microphase separation of Azo and PEG and PEG crystallization, coexisted in the system. We further increased the Azo content to 50% (Figure 9d). The two peaks representing the microphase separation of Azo and PEG were observed for the entire temperature range of the measurement. Using the one-dimensional correlation function [49], we obtained the average long period of 34.7 nm, which was identical to the interlamellar distance obtained from the first-order peak position of the SAXS profile. The mean thicknesses of the Azo-rich and PEG-rich microphases were 11.5 and 23.2 nm, respectively. Furthermore, the peaks that were related to PEG crystallization did not appear in the 50:50 Azo/PEG complex, which is consistent with the DSC and WAXS results that indicated PEG crystallization was suppressed by the addition of Azo. These results suggested that microphase separation occurred only when the Azo content was sufficiently high. Additionally, microphase separation was not induced by PEG crystallization.

**Figure 10:**
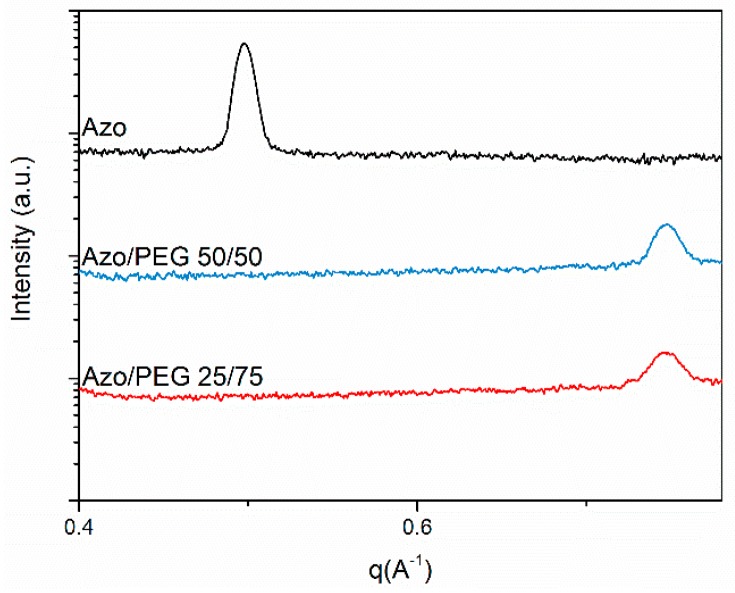
SAXS patterns of PEG and its complexes in combination with Azo at low *q* regions (measured at room temperature). The complex compositions are designated in wt %.

The SAXS patterns in the small *q* regions present the long-range order associated with PEG crystallization and microphase separation of PEG and Azo. The arrangement of azobenzene mesogens is considered to be at the smaller length scale, which was beyond the q range measured, as depicted in Figure 8. Therefore, we conducted the SAXS measurements of Azo/PEG complexes at *q* values of 0.40–0.85 Å^−1^, as seen in Figure 10. The SAXS pattern of the neat Azo exhibited a single peak at the q value of 0.498 Å^−1^, which resulted from the periodic ordering of mesogens. The lack of higher order peaks indicated that mesogens formed only short-range orders. The long period calculated by Bragg’s law was approximately 12.6 Å. This value was slightly smaller the trans-azobenzene length of approximately 15.0 Å [50], indicating the formation of tilted monosmectic layers of mesogens with a tilt angle to the layer normal. The tilt angle can be calculated by cos^−1^ (long period/15.0), and a tilt angle of 32.9° was obtained. When Azo was incorporated into PEG, the peak at the *q* value of 0.498 Å^−1^ disappeared. Instead, a peak at the q value of 0.746 Å^−1^ was obtained, which corresponded to the long period of 8.4 Å and the tilt angle of 55.9°. The long period of Azo/PEG complexes was smaller than that of Azo, and this suggested that the formation of complexes through hydrogen bonds facilitated the compact packing of mesogens.

**Figure 11:**
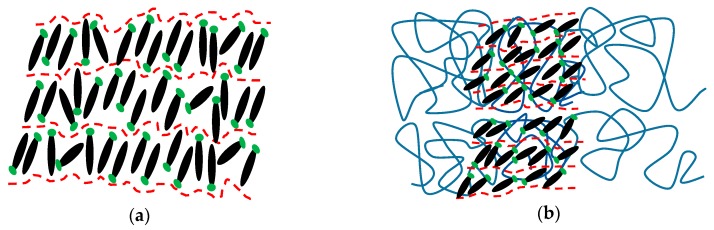

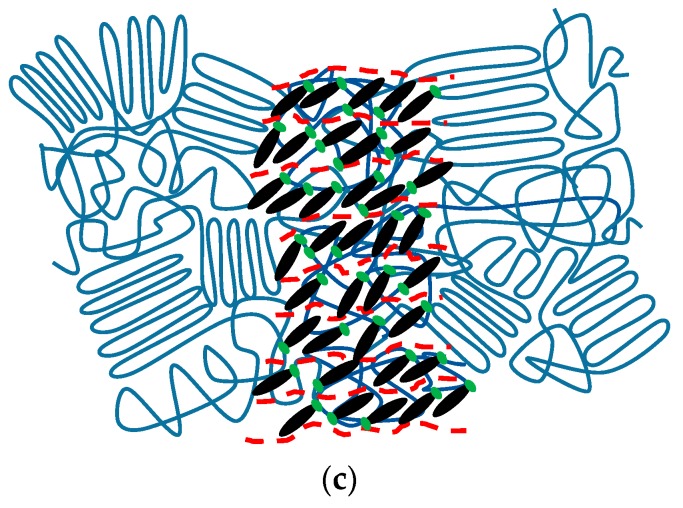
Phase behavior of Azo/PEG complexes: (**a**) Azo, (**b**) Azo/PEG before crystallization, and (**c**) Azo/PEG after crystallization.

Figure 11 demonstrates the schematics of the self-assembled structures formed by Azo and PEG through hydrogen-bonding interaction. In the neat Azo, the van der Waals interaction between mesogens acted as a driving force, resulting in side-by-side and head-to-head packed mesogens. However, the van der Waals interaction resulted only in a short-range order of mesogens, forming a poorly ordered lamellar structure, as seen in Figure 11a. When PEG was complexed with Azo, the hydrogen-bonding interaction improved the regularity of the complex, promoting the self-assembly of the constituents into a well-ordered lamellar morphology composed of PEG-rich and Azo-rich microphases. This microphase-separated lamellar morphology resembles the supramolecular materials formed by a homopolymer and a small molecule with hydrogen bonds [51]. By contrast, mesogens confined in the Azo-rich microphases organized into a lamellar structure, producing lamellae containing Azo within lamellar phase morphology (Figure 11b). Because of the two independent series of diffraction peaks in SAXS patterns, the normals of the two different length-scale lamellae were not aligned in the same direction. The confinement of mesogens in the Azo-rich microphases facilitated the aggregation of mesogens, resulting in increased quantities of H-aggregated and J-aggregated mesogens. Furthermore, when PEG was crystallized, the lamellar phase morphology of the complex did not change, indicating that PEG crystallization occurred within hard confinement (Figure 11c). The hard confinement hampered the formation of chain-folded crystals, causing decreases in the crystallization temperature and degree of crystallinity. Consequently, PEG crystallization was prohibited when the PEG content was 50%.

## 4. Conclusions

We demonstrated the structure and photoresponsive and crystallization behaviors of complexes composed of Azo and PEG. In the complexes, Azo and PEG formed hydrogen bonds, improving the miscibility of the components. The resulting complexes exhibited photoresponsive behavior in solvents, but the photoisomerization rate of complexes reduced compared with that of neat Azo. Such behavior was attributed to the hydrogen-bonding interaction between Azo and PEG that hindered the rotation and inversion of mesogens, thereby causing a reduction of the photoisomerization rate. When Azo/PEG complexes were prepared in thin films, the confined environment resulted in a substantial decrease in the photoisomerization rate. Additionally, the amounts of H-aggregated and J-aggregated mesogens increased with an increase in the PEG content. Adding Azo to PEG considerably decreased the crystallization temperature and degree of crystallinity of PEG, which were associated with the hydrogen-bonding interaction between Azo and PEG that suppressed PEG crystallization. UV irradiation of Azo/PEG complexes resulted in an increase in the crystallization temperature. This was attributed to the presence of high-polarity cis isomers after photoisomerization, which improved compatibility between mesogens and PEG. Furthermore, the complexation of Azo and PEG induced microphase separation, forming a lamellar morphology. In the Azo-rich microphases, the confined regions resulted in tilted monosmectic layers of mesogens. PEG crystallization did not alter the domain size of the lamellar morphology, suggesting that PEG crystallization was hard confined within preexisting Azo glassy layers. PEG crystallization in lamellar microphases was prohibited for the PEG content of up to 50%.

## Figures and Tables

**Table 1:** Rate constants of trans-to-cis photoisomerization for neat Azo and for its complex in combination with PEG in various solvents obtained through the UV–Vis spectrum.

Azo/PEG (wt %/wt %)	Rate Constant (s^−1^)			
	Benzene	Toluene	THF	CH_2_Cl_2_
100/0	0.140	0.139	0.133	0.118
50/50	0.083	0.099	0.132	0.089

**Table 2:** Thermal and crystallization behaviors of Azo/PEG complexes.

Azo/PEG	*T*_c_ (°C)	*T*_m_ (°C)	Degree of Crystallinity (%)
0/100	37.3	59.6	85.4
10/90	35.8	55.6	70.1
10/90 with UV exposure	36.2	56.0	74.7
25/75	26.0	51.5	63.4
25/75 with UV exposure	27.1	51.6	66.4
38/62	13.9	44.2	30.2
38/62 with UV exposure	19.9	44.6	31.0
